# Recent Advances in Applied Microbiology: Editorial

**DOI:** 10.3390/microorganisms8091364

**Published:** 2020-09-07

**Authors:** Letícia M. Estevinho, Patrícia Combarros-Fuertes, Vanessa Branco Paula

**Affiliations:** 1 Centro de Investigação de Montanha (CIMO), Instituto Politécnico de Bragança, 5300-252 Bragança, Portugal; vanessapaula@ipb.pt; 2 Department of Food Hygiene and Technology, Faculty of Veterinary Science, University of León, Campus de Vegazana, 24071 León, Spain; pcomf@unileon.es

**Keywords:** agricultural microbiology, applied microbiology, food microbiology, industrial microbiology, medical microbiology

## Abstract

The importance of microbiology has grown exponentially since the development of genomics, transcriptomics, and proteomics, making it possible to clarify microbial biogeochemical processes and their interactions with macroorganisms in both health and disease. Particular attention is being payed to applied microbiology, a discipline that deals with the application of microorganisms to specific endeavors, whose economic value is expected to exceed USD 675.2 billion by 2024. In the Special Issue “Recent Advances in Applied Microbiology”, twenty-four papers were published (four reviews and twenty original research papers), covering a wide range of subjects within applied microbiology, including: microbial pathogenesis, the health-promoting properties of microorganisms and their by-products, food conservation, the production of alcoholic beverages, bioremediation and the application of microbiology to several industrial processes.

Microorganisms, their activity and metabolites have remarkable effects on the functioning of human beings and the entire biological world, being the life-support system of the whole biosphere [1]. Microbiology, the science exploring the interactions between micro- and macro-organisms, both in health and disease, is a branch of life sciences that has grown exponentially since the establishment of genomics, transcriptomics, and proteomics. Applied microbiology is the discipline that deals with the application of microorganisms to specific endeavours, including: the optimization of animal and plant crops, the production of foods and supplements, the production of chemicals and biomaterials trough fermentation, the recovery of natural resources and energy production, the treatment of waste and the bioremediation of polluted sites [2], the production of drugs, vaccines, diagnostic tools and biosensor systems, the development of microbial therapies for dysbiosis-related diseases, and the microbial-induced modulation of biotechnological relevant cells and organisms [3].

The Special Issue entitled “Recent Advances in Applied Microbiology” aimed to document and increase the visibility of the current discoveries on this vast field. Twenty-four papers were published (four reviews and twenty original research papers) from teams situated in Asia, Europe, North America and South America, demonstrating the global impact of this thematic.

The reviews covered very pertinent topics. Malavia et al. [4] focused on human fungal pathogenesis by exploring the advantages and limitations of different molecular tools (interference ribonucleic acid, CRISPR technologies and transposon targeted mutagenesis) and in vivo models (zebrafish, the silkworm and murine), opening doors for improvements in the experimental designs of future studies. Wang et al. [5] developed an overview of the advances in the production of fungal laccase (a copper-containing oxidase enzyme) using lignocellulosic agricultural wastes. Among other aspects, this study emphasized the selection of subtracts and fungal strains and compared the traditional submerged and the solid-state fermentations. The review by Kobayashi et al. [6] described the d-amino acids produced by lactic acid bacteria. Besides being important for the flavour characteristics of lactic-acid-fermented foods, authors reported that those amino-acids have functional roles (e.g., in increasing collagen and preventing cells’ oxidation). Another team provided an update on the research on diatoms, a fundamental source of carbon, using a very interesting methodology—latent dirichlet allocation (LDA), a generative model that identifies the investigation trends in a given topic [7].

The original research articles published also covered a wide range of sectors. Across the field of medical microbiology, an innovative study explored the immunomodulatory properties of a mixture of lactic acid bacteria with probiotic features in an in vitro model of macrophage-like cells and a chemically induced colorectal cancer model [8]. Another interesting study reported the hepatoprotective properties of dietary *Clostridium butyricum*; such effects were mediated by the modulation of genes and pathways associated with immunoregulation and lipid metabolism [9]. Combarros-Fuertes et al. [10] analysed the mechanisms underlying the antibacterial activity of manuka honey; it induced metabolic disruption in *Staphylococcus aureus* and was able to block efflux pump activity in a dose-dependent fashion in *Escherichia coli.* Also within this scope, Rocha et al. [11] analysed the diversity of the endophytic fungi produced by the medicinal plant *Schinus terebinthifolius*, the chemical compounds produced by the endophytes, and their antibacterial and antioxidant activities.

Concerning the topic of food microbiology, Cao et al. [12] described, for the first time, the promising characteristics of encapsulated *Bdellovibrio* powder as a bio-disinfectant against whiteleg shrimp-pathogenic vibrios. Regarding the production of alcoholic beverages, Pereira et al. [13] studied the volatile composition and sensory properties of mead produced using free and immobilized yeasts. Another Portuguese study by Esteves et al. [14] provided evidence that non-conventional yeast *Saccharomycodes ludwigii* has potential for wine production, either in monoculture fermentation or as a co-starter culture with *Saccharomyces cerevisiae.*

Within the scope of agricultural microbiology, Xu et al. [15] evaluated the effects of eucalyptus age and species on the soil microbial biomass and enzyme activities. In addition, two studies, performed by the same group, intended to explore the diversity and characteristics of rumen bacterial community [16], the digestive ability and physiological characteristics of finishing cattle after dietary changes [17].

Three studies provided advances in the field of bioremediation. Fang et al. [18] studied three denitrifying bacteria and two functional genes as potential biomarkers for total nitrogen removal. Cai et al. [19] used metagenomics analysis to explore the biodegradation related metabolism in an abnormally low dissolved inorganic carbon petroleum-contaminated aquifer. Algal biomass degradation by marine fungi isolated from the brown seaweed *Fucus* sp. was also studied [20]. Another team focused the production of biodiesel using *E. coli* with an engineered overexpressed fatty acid operon [21].

Within the framework of industrial bioprocesses, Gomes et al. [22] assessed the production of hyaluronic acid using *Kluyveromyces lactis* following the addition of the genes *hasA* and *hasB* (from *Pasteurella multocida and Xenopus leavis*, respectively). The experiments carried out by Lee et al. [23], using *Deinococcus geothermalis,* uncovered the pathway towards a better understanding of the physiological pros and cons of transposition phenomena. A German team investigated glucose-6-phosphate dehydrogenase genes expressed heterologously in a zwf1 deletion yeast. Considering the major role of those genes in human diseases and in plant growth, such systems are expected to possess a broad range of applications [24]. In another publication, Feng et al. [25], using a multilayer dielectric model of filamentous fugal cells, provided theoretical support for applying high-voltage pulsed electric fields to kill fungi, which may be useful for several practical applications. *Aspergillus oryzae* is an important industrial microorganism for the production of traditional fermented products and enzymes; the report of Shao et al. [26] provides innovative information regarding the responses of this filamentous fungi against various oxidative stresses. Last but not the least, a Korean group used *Pseudomonas fluorescens* to produce bacteriotoxic phospholipase A1, which is useful to efficiently degum oil [27].

## References

[1] Timmis K., Cavicchioli R., Garcia J.L., Nogales B., Chavarría M., Stein L., McGenity T.J., Webster N., Singh B.K., Handelsman J. (2019). The urgent need for microbiology literacy in society. Environ. Microbiol..

[2] Wang J., Yin Y. (2019). Progress in microbiology for fermentative hydrogen production from organic wastes. Crit. Rev. Environ. Sci. Technol..

[3] Miller M.B., Atrzadeh F., Burnham C.A., Cavalieri S., Dunn J., Jones S., Mathews C., McNult P., Meduri J., Newhouse C. (2019). Clinical Utility of Advanced Microbiology Testing Tools. J. Clin. Microbiol..

[4] Malavia D., Gow N.A.R., Usher J. (2020). Advances in Molecular Tools and In Vivo Models for the Study of Human Fungal Pathogenesis. Microorganisms.

[5] Wang F., Xu L., Zhao L., Ding Z., Ma H., Terry N. (2019). Fungal Laccase Production from Lignocellulosic Agricultural Wastes by Solid-State Fermentation: A Review. Microorganisms.

[6] Kobayashi J. (2019). d-Amino Acids and Lactic Acid Bacteria. Microorganisms.

[7] Zhang Y., Tao J., Wang J., Ding L., Ding C., Li Y., Zhou Q., Li D., Zhang H. (2019). Trends in Diatom Research Since 1991 Based on Topic Modeling. Microorganisms.

[8] Hradicka P., Beal J., Kassayova M., Foey A., Demeckova V. (2020). A Novel Lactic Acid Bacteria Mixture: Macrophage-Targeted Prophylactic Intervention in Colorectal Cancer Management. Microorganisms.

[9] Liu Y., Liu C., Huang L., Xia Z. (2019). A Discovery of Relevant Hepatoprotective Effects and Underlying Mechanisms of Dietary Clostridium butyricum Against Corticosterone-Induced Liver Injury in Pekin Ducks. Microorganisms.

[10] Combarros-Fuertes P., Estevinho L.M., Teixeira-Santos R., Rodrigues A.G., Pina-Vaz C., Fresno J.M., Tornadijo M.E. (2019). Evaluation of Physiological Effects Induced by Manuka Honey Upon *Staphylococcus aureus* and *Escherichia coli*. Microorganisms.

[11] Santos da Rocha P.d., Paula V.M.B., Olinto S.C.F., dos Santos E.L., de Picoli Souza K., Estevinho L.M. (2020). Diversity, Chemical Constituents and Biological Activities of Endophytic Fungi Isolated from Schinus terebinthifolius Raddi. Microorganisms.

[12] Cao H., Wang H., Yu J., An J., Chen J. (2019). Encapsulated Bdellovibrio Powder as a Potential Bio-Disinfectant against Whiteleg Shrimp-Pathogenic Vibrios. Microorganisms.

[13] Pereira A.P., Mendes-Ferreira A., Dias L.G., Oliveira J.M., Estevinho L.M., Mendes-Faia A. (2019). Volatile Composition and Sensory Properties of Mead. Microorganisms.

[14] Esteves M., Barbosa C., Vasconcelos I., Tavares M.J., Mendes-Faia A., Pereira Mira N., Mendes-Ferreira A. (2019). Characterizing the Potential of the Non-Conventional Yeast Saccharomycodes ludwigii UTAD17 in Winemaking. Microorganisms.

[15] Xu J., Liu B., Qu Z.-L., Ma Y., Sun H. (2020). Age and Species of Eucalyptus Plantations Affect Soil Microbial Biomass and Enzymatic Activities. Microorganisms.

[16] Qiu Q., Gao C., Gao Z., He Y., Cao B., Su H. (2019). Temporal Dynamics in Rumen Bacterial Community Composition of Finishing Steers during an Adaptation Period of Three Months. Microorganisms.

[17] Qiu Q., Gao C., Aziz Ur Rahman M., Cao B., Su H. (2020). Digestive Ability, Physiological Characteristics, and Rumen Bacterial Community of Holstein Finishing Steers in Response to Three Nutrient Density Diets as Fattening Phases Advanced. Microorganisms.

[18] Fang H., Olson B.H., Asvapathanagul P., Wang T., Tsai R., Rosso D. (2019). Molecular Biomarkers and Influential Factors of Denitrification in a Full-Scale Biological Nitrogen Removal Plant. Microorganisms.

[19] Cai P., Ning Z., Zhang N., Zhang M., Guo C., Niu M., Shi J. (2019). Insights into Biodegradation Related Metabolism in an Abnormally Low Dissolved Inorganic Carbon (DIC) Petroleum-Contaminated Aquifer by Metagenomics Analysis. Microorganisms.

[20] Patyshakuliyeva A., Falkoski D.L., Wiebenga A., Timmermans K., de Vries R.P. (2019). Macroalgae Derived Fungi Have High Abilities to Degrade Algal Polymers. Microorganisms.

[21] Rahman Z., Sung B.H., Nawab J., Siddiqui M.F., Ali A., Geraldi A., Kim S.C. (2019). Enhanced Production of Fatty Acid Ethyl Ester with Engineered fabHDG Operon in *Escherichia coli*. Microorganisms.

[22] Gomes A.M., Netto J.H.C.M., Carvalho L.S., Parachin N.S. (2019). Heterologous Hyaluronic Acid Production in Kluyveromyces lactis. Microorganisms.

[23] Lee C., Choi N., Bae M.K., Choo K., Lee S.-J. (2019). Transposition of Insertion Sequences was Triggered by Oxidative Stress in Radiation-Resistant Bacterium Deinococcus geothermalis. Microorganisms.

[24] Heinisch J.J., Knuesting J., Scheibe R. (2020). Investigation of Heterologously Expressed Glucose-6-Phosphate Dehydrogenase Genes in a Yeast zwf1 Deletion. Microorganisms.

[25] Feng X., Zhu M., Xu J., Yin W., Hu F. (2019). Analysis of Factors Influencing the Transmembrane Voltage Induced in Filamentous Fungi by Pulsed Electric Fields. Microorganisms.

[26] Shao H., Tu Y., Wang Y., Jiang C., Ma L., Hu Z., Wang J., Zeng B., He B. (2019). Oxidative Stress Response of Aspergillus oryzae Induced by Hydrogen Peroxide and Menadione Sodium Bisulfite. Microorganisms.

[27] Park J., Eom G.T., Young Oh J., Hyun Park J., Kim S.C., Kwang Song J., Hoon Ahn J. (2020). High-Level Production of Bacteriotoxic Phospholipase A1 in Bacterial Host Pseudomonas fluorescens Via ABC Transporter-Mediated Secretion and Inducible Expression. Microorganisms.

